# Mapping Connectivity Amongst Interneuronal Components of the Locomotor CPG

**DOI:** 10.3389/fncel.2019.00443

**Published:** 2019-10-04

**Authors:** Farhia Haque, Simon Gosgnach

**Affiliations:** ^1^Neuroscience and Mental Health Institute, University of Alberta, Edmonton, AB, Canada; ^2^Department of Physiology, Faculty of Medicine & Dentistry, University of Alberta, Edmonton, AB, Canada

**Keywords:** locomotion, central pattern generator, interneuron, synapse, connectivity

## Abstract

The basic rhythmic activity characteristic of locomotion in mammals is generated by a neural network, located in the spinal cord, known as the locomotor central pattern generator (CPG). Although a great deal of effort has gone into the study of this neural circuit over the past century, identification and characterization of its component interneurons has proven to be challenging, largely due to their location and distribution. Recent work incorporating a molecular approach has provided a great deal of insight into the genetic identity of interneurons that make up this neural circuit, as well as the specific roles that they play during stepping. Despite this progress we still know relatively little regarding the manner in which these neuronal populations are interconnected. In this article we review the interneuronal populations shown to be involved in locomotor activity, briefly summarize their specific function, and focus on experimental work that provides insight into their synaptic connectivity. Finally, we discuss how recently developed viral approaches can potentially be incorporated to provide further insight into the network structure of this neural circuit.

## Introduction

When broken down into its individual movements, the act of walking is an alarmingly complex activity which requires the precise contraction of numerous muscles on either side of the body, while cortical and sensory information is continuously processed and integrated in order to generate seamless, fluid movement. While in the intact animal, locomotor activity is initiated by descending input, the basic alternation that is characteristic of mammalian locomotion is generated by a neural circuit that is located in the spinal cord known as the locomotor central pattern generator (CPG- reviewed in Kiehn, 2016). Once activated, this neural circuit is independently able to generate long lasting locomotor activity and, remarkably, is also able to modulate its output to account for complex sensory information (Forssberg, 1979; Quevedo et al., 2005; Rossignol et al., 2006).

Although the mammalian locomotor CPG was discovered more than a century ago (Brown, 1911), progress toward identifying the interneuronal components of this neural network has been relatively slow. Recent advances in molecular genetic, anatomical tracing, and imaging techniques have generated a substantial amount of new information regarding components of the mammalian locomotor CPG. At the spinal level, a molecular approach has been used to divide the developing neural tube of the embryonic mouse into ten distinct “parent” populations of interneurons (dI1-dI6, V0-V3) based on transcription factor expression (Goulding and Lamar, 2000; Goulding, 2009; Arber, 2012; Lu et al., 2015). Subsequent characterization of each population has resulted in the identification of a number of distinct subpopulations within each “parent” population based on downstream transcription factor expression (Catela et al., 2015; Gosgnach et al., 2017; Ziskind-Conhaim and Hochman, 2017; Cote et al., 2018; Bikoff, 2019). Since transcription factor expression dictates neuronal characteristics such as cell fate, channel composition, axonal projection pattern, and neurotransmitter phenotype, it was originally postulated that populations of neurons with a similar genetic background would have similar characteristics, and a similar function during locomotor activity. Studies performed over the past 15 years have characterized each of the genetically-defined neuronal populations that settle in the spinal cord, and defined their function during locomotor activity (reviewed in Grossmann et al., 2010; Kiehn, 2016; Deska-Gauthier and Zhang, 2019). Based on this work we can now identify the specific populations that are responsible for such key functions as left-right (Lanuza et al., 2004; Talpalar et al., 2013), and flexor-extensor (Zhang et al., 2014; Britz et al., 2015) coordination.

Despite this progress we still know very little regarding the manner in which these populations are interconnected to one another in order to produce locomotor activity. This is essential information if we are to gain a better understanding of the structure and mechanism of function of the locomotor CPG. In this article we will discuss the current state of knowledge regarding the synaptic connectivity of each population that has been shown to be involved in locomotor activity. Although computational modeling has been used to predict synaptic connectivity amongst many of these populations (Rybak et al., 2015; Shevtsova et al., 2015; Danner et al., 2016, 2017; Shevtsova and Rybak, 2016) we will not discuss this work here, we will simply provide a comprehensive summary of the findings of anatomical tracing and electrophysiological experiments that have probed connectivity amongst neuronal components of the locomotor CPG situated in the lumbar spinal cord. Finally, we will present new data which identifies the upstream synaptic partners of WT1-expressing interneurons, and discuss recently developed viral approaches that can be utilized to provide insight into the connectivity of interneuronal populations that comprise the locomotor CPG and generate key information regarding the network structure of this neural circuit.

## V0 Interneurons

The V0 population can be divided up into a dorsal subpopulation (V0_D_) which expresses the transcription factor Dbx1 postmitotically, and a ventral population (V0_V_) that expresses Evx1 in addition to Dbx1 (Moran-Rivard et al., 2001; Pierani et al., 2001). Both the V0_D_ and V0_V_ subsets are intermingled in lamina VIII of the postnatal spinal cord.

Initial studies into the function of the V0 population during locomotor activity indicated that these cells are crucial for appropriate alternation of contralateral motoneurons during stepping (Lanuza et al., 2004). A subsequent study considered the specific role of the V0_D_ and V0_V_ subpopulations independently, and demonstrated that the dorsal subset is inhibitory and responsible for coordinating left-right alternation at slow locomotor speeds, while the ventral subset is excitatory and responsible for this function when locomotor speed increases (Talpalar et al., 2013; Bellardita and Kiehn, 2015).

The specific connectivity responsible for this speed-dependent control of left-right alternation during locomotion has not been demonstrated. Investigations into the axonal projection pattern of V0 neurons located in the lumbar spinal cord indicated that greater than 90% of the axons from both V0_D_ and V0_V_ cells project to the contralateral spinal cord and, after crossing the midline, project up to four segments in the rostral direction (Moran-Rivard et al., 2001; Pierani et al., 2001). V0_V_ neurons in the cervical spinal cord have been shown to project long descending commissural axons which terminate on neurons in the lumbar segments (Ruder et al., 2016). Retrograde transynaptic tracing with pseudorabies virus, which expresses GFP in all infected cells (i.e., PRV-152- Kerman et al., 2003), demonstrated that the V0 neurons in the lumbar segments contact contralateral motoneurons (Lanuza et al., 2004), although it is essential to keep in mind that V0 cells may synapse onto additional cell types since it is not possible to identify downstream synaptic partners other than motoneurons using this experimental approach, nor is it possible to determine the proportion of V0 neurons that contact motoneurons.

Additional analysis of the genetic lineage of the V0 population has indicated that the Evx1-expressing V0_V_ subpopulation contains a small number of cells (5% of the entire V0 population) that express the transcription factor Pitx2, are cholinergic, project axons both ipsilaterally and contralaterally, and make contact onto motoneurons as well as unidentified interneurons in the dorsal and intermediate nucleus of the spinal cord (Zagoraiou et al., 2009). These neurons are referred to as the V0_C_ subset and, based on functional deficits which are apparent in their absence, they have been hypothesized to modulate motoneuronal activity in a task dependent manner during stepping (Zagoraiou et al., 2009).

## V1 Interneurons

The V1 neurons are overwhelmingly inhibitory and primarily located in lamina VII/IX of the spinal cord (Saueressig et al., 1999). The Renshaw cells, a functionally defined population which receives input from motoneurons and inhibits Ia inhibitory interneurons (IaINs) and motoneurons (Renshaw, 1941; Eccles et al., 1954), as well as the Ia INs, which receive input from muscle afferents and Renshaw cells and inhibit motoneurons (Mendell and Henneman, 1971), are each derived from this cell population (Alvarez et al., 2005; Stam et al., 2012). Outside of these two subsets, which together comprise 22% of the entire population, V1 neurons exhibit a tremendous amount of diversity. Recent work demonstrated that a minimum of 19 genetically distinct “clades” of V1 neurons, each with a unique physiological signature and distribution in the ventral spinal cord (Bikoff et al., 2016; Gabitto et al., 2016).

Given the diversity inherent within this population it is not surprising that a number of functions have been attributed to the V1 neurons. Ablation or silencing of the entire V1 population results in a drastic slowing of the locomotor rhythm (Gosgnach et al., 2006), while ablation of both the V1 and V2b neurons results in aberrant ipsilateral flexor-extensor alternation, and indicates that these two populations work together to coordinate the activity of ipsilateral motoneurons during stepping (Zhang et al., 2014). These latter results match up well with anatomical data showing that V1 neurons preferentially synapse on flexor motoneurons (Britz et al., 2015) while V2b neurons primarily contact extensor motoneurons. In addition, to contacting motoneurons, terminals from V1 neurons can also be found in close proximity to unidentified interneurons in lamina VII as well as the deep dorsal horn (Alvarez et al., 2005).

Given the molecular and physiological diversity displayed by this population it is essential to keep in mind that inhibition or deletion of the entire V1 population results in loss of function of multiple “clades,” each of which likely possess a more discreet function during stepping, and the locomotor phenotypes observed when the entire population is silenced or ablated may be a consequence of the removal of several, functionally heterogeneous subtypes.

## V2 Interneurons

The V2 population can be divided up into the V2a subset, which are excitatory and express the transcription factor Chx10, as well as the inhibitory V2b subset which express GATA2/3 (Lundfald et al., 2007; Peng et al., 2007). While both subsets of V2 neurons are primarily located in laminae VII-X of the ventral spinal cord postnatally, there is a subtle variation in their distribution with the V2a cells evenly distributed throughout laminae VII, VIII, and X, while the V2b cells are clustered in lamina X and lamina IX (Lundfald et al., 2007). Both the V2a and V2b neurons located in the lumbar spinal cord project their axons exclusively to the ipsilateral side of the spinal cord before terminating within two segments in the rostral and caudal directions (Lundfald et al., 2007). Consistent with their aforementioned role in coordinating flexor-extensor alternation during locomotion, the V2b neurons preferentially contact extensor (rather than flexor) motoneurons, and synaptic terminals from this subset have also been found on members of the V0_C_ population as well as unidentified neurons in lamina VII/VIII (Zhang et al., 2014). In addition, subsequent study of the V2a (Ruder et al., 2016) and V2b (Flynn et al., 2017) populations located in the cervical spinal cord demonstrated that both subtypes are involved regulating hindlimb locomotor activity via long descending ipsilateral connections to neurons located in the lumbar spinal cord.

Surprisingly, given their strictly ipsilateral axonal projection pattern, the V2a neurons were shown to be involved in coordinating left-right alternation during locomotor activity (Crone et al., 2008). This was hypothesized to be mediated by excitation of commissural interneurons which were shown to receive input from the V2a population (Crone et al., 2008). While the genetic background of a significant portion of these commissural neurons is unknown, the finding that a subset belong to the Evx1-expressing V0_V_ interneurons is consistent with later work indicating that the V2a neurons are dispensable for slow locomotor activity but required for left-right alternation as the frequency of stepping increases (Crone et al., 2009). While there is no direct experimental evidence indicating that V2a neurons excite ipsilateral motoneurons, this connectivity has been predicted since the burst amplitude of motor axons varies during locomotor activity when Chx10 neurons are absent (Dougherty and Kiehn, 2010).

## V3 Interneurons

The V3 interneurons express the transcription factor Sim1 (Briscoe et al., 1999), and are spread through the dorsal, intermediate, and ventral spinal cord at birth (Zhang et al., 2008). Recent electrophysiological characterization of this population has demonstrated that V3 cells in each of these regions possesses unique electrophysiological characteristics (Borowska et al., 2013, 2015). Overall, 97% of V3 neurons project commissural axons (Blacklaws et al., 2015), and investigation of their neurotransmitter phenotype indicated that this population is exclusively excitatory (Zhang et al., 2008).

Regarding their specific synaptic partners, injection of the retrograde transsynaptic tracer PRV-152 into the hindlimb musculature of mice indicated that at least a subset of V3 cells contact motoneurons, with approximately 90% of the V3 cells with this projection pattern contacting motoneurons on the contralateral side of the spinal cord. As previously discussed, tracing with PRV-152 does not enable the specific proportion of the population that contacts motoneurons to be determined, and we know that V3 neurons also contact IaINs, Renshaw cells, as well as V2b cells and other, unidentified, commissural neurons (Zhang et al., 2008). Recent work incorporating an electrophysiological approach demonstrated that some of these commissural neurons which receive input from the V3 neurons may also belong to the V3 population themselves. Photostimulation of ventromedially located V3 cells was shown to activate other neighboring V3 cells as well as those in the ventrolateral spinal cord, while photostimulation of ventrolaterally located V3 cells was shown to excite ipsilateral motoneurons (Chopek et al., 2018). Many of the ventromedially and ventrolaterally located V3 neurons were also shown to have a contralaterally projecting axonal branch, however the specific termination point, and functional impact of this branch is unknown (Chopek et al., 2018). At this point the specific role of each subpopulation of V3 cells during locomotion is unclear. All functional studies have been carried out in the absence of the entire V3 population which has led to non-specific locomotor deficits such as a lack of robustness and regularity, indicative of a population that may have a number of functionally diverse subsets (Zhang et al., 2008). A more complete picture of the role of V3 neurons during locomotor activity will be apparent once each subsets of this population has been identified and investigated individually.

## dI6 Interneurons

dI6 neurons express the transcription factors WT1 or DMRT3 at postmitotic time points (Goulding, 2009), and settle in lamina VII/VIII of the postnatal spinal cord (Dyck et al., 2012; Griener et al., 2017). The DMRT3 subset of dI6 neurons is an inhibitory population (Perry et al., 2019) which projects axons to both ipsilateral and contralateral targets. Retrograde tracing with PRV-152 demonstrated that the DMRT3 neurons project to motoneurons on either side of the spinal cord (Andersson et al., 2012) but again, given the nature of these experiments, the proportion of the DMRT3 population with this projection pattern is unknown. Recent work has also identified axon terminals from DMRT3-expressing neurons in close proximity to Renshaw cells as well as cholinergic neurons (possibly V0_C_ interneurons) located near the central canal (Perry et al., 2019). This population has been predicted to play a critical role during locomotion based on data indicating that they are rhythmically active during stepping (Perry et al., 2019), as well as defects observed in left-right alternation in their absence (Andersson et al., 2012).

Like the DMRT3-expressing subset of dI6 cells, the WT1+ neurons are inhibitory. Work carried out in my laboratory has demonstrated that this subset extends commissural axons (Haque et al., 2018), however they do not make monosynaptic contact onto motoneurons. Synaptic terminals of WT1+ neurons were observed in close proximity to commissural neurons in the intermediate spinal cord, specifically members of the DMRT3+ and Evx1+ populations (Haque et al., 2018). All WT1-expressing neurons recorded during fictive locomotion were rhythmically-active and their importance during locomotion was confirmed by experiments which demonstrated left-right coordination defects were apparent when this subpopulation is silenced (Haque et al., 2018). Based on their connectivity as well as the locomotor phenotype in their absence we postulated that they work together with the V0 and DMRT3 populations to regulate left-right alternation during stepping (Haque et al., 2018).

**FIGURE 1:**
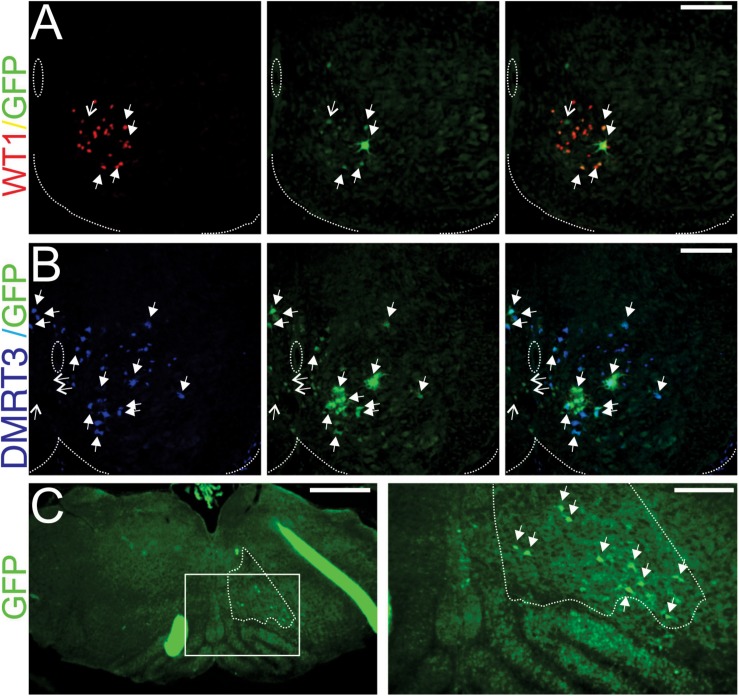
WT1-expressing neurons in the lumbar spinal cord receive monosynaptic input from DMRT3+ cells and well as the reticular formation. **(A)** 20 μm section cut from the third lumbar (L3) spinal segment of a WT1^*Cre*^ mouse 20 h after Introvert-PRV injection reveals that the majority of cells taking up the virus (green cells, GFP+) express WT1 (red cells). Filled arrows indicate infected cells that are WT1+, open arrows indicate those that are WT1−. **(B)** 20 μm section cut from the L2 spinal segment 30 h after microinjection of Introvert-PRV into the L3 segment illustrates that the some DMRT3+ neurons (blue) are infected (green) and thus monosynaptically connect to WT1-expressing neurons. Filled arrows indicate infected cells that are DMRT3+ and open arrows those that are DMRT3-. In panels **(A,B)** dashed lines indicate the central canal and the ventral extent of the spinal cord, scale Bars = 100 μm. **(C)** Section cut from the brainstem 30 h after injection of Introvert PRV into the L3 segment reveal that cells within the reticular nucleus gigantocellularis (region surrounded by dashes, determined by consultation with the Allen Brain Atlas) are infected (green cells indicated by arrows in panel to the right) and thus contact WT1 neurons in the lumbar spinal cord. Panel to the right is a magnification of boxed region in the left panel. Scale bar in the left panel = 500 μm, in the right panel 200 μm.

Further support for this hypothesis comes from recent work from our laboratory in which we incorporated a viral approach to investigate the upstream synaptic partners of WT1-expressing neurons. All procedures for these experiments were performed on mice in accordance with the Canadian Council on Animal Welfare and approved by the Animal Welfare Committee at the University of Alberta. For these experiments four 3–5 days old WT1^*Cre*^ mice of either sex were anesthetized with isoflurane, and once surgical plane was reached a small incision was made directly over the L3 segment, a laminectomy was performed at this segment, and 100–200 nL of the Cre-dependent, retrograde, transsynaptic virus Introvert Pseudorabies virus (i.e., Introvert-PRV, kind gift Dr. Jeffrey Friedman, HHMI, Rockefeller Univ.) was microinjected directly into the ventral aspect of the third lumbar spinal segment (i.e., L3). This virus has been shown to infect, and express GFP, in Cre expressing cells at the injection site between 15 and 20 h after injection, before traveling to all monosynaptically connected upstream synaptic partners 26–30 h after injection (Pomeranz et al., 2017). At the appropriate time after injection animals were anesthetized and perfused with 4% paraformaldehyde (PFA). The entire CNS was dissected out, and tissue was post fixed (24 h in 4% PFA), cryoprotected in 30% sucrose, and frozen in preparation for cryosectioning. Inspection of sections cut 20 h after injection and stained with antibodies to GFP (to identify cells that took up the virus) and WT1 revealed that 87% of GFP- expressing neurons were WT1+ (Figure 1A) confirming that this virus preferentially infects Cre expressing neurons.

To determine the identity of the spinal neurons that synapse onto WT1-expressing cells sections cut from spinal cords harvested 30 h post injection were stained with antibodies against Evx1, En1, Chx10, and Dmrt3 in order to label V0_V_, V1, V2a, and a subset of the dI6 interneuronal populations, respectively. A mean of 5.4 ± 3.9 (SD) GFP+ (i.e., viral infected) cells per 20 μm hemi-section (*n* = 5) were found throughout the lower thoracic to lumbar (T10-L5) segments of the spinal cord. We found no co-labeling with En1 or Chx10- expressing neurons, and only one viral infected cell that expressed Evx1 (i.e., a V0_V_ neuron), however 95.9% of viral infected cells were DMRT3+ (Figure 1B), indicating that much of the input to WT1-expressing neurons at the spinal level comes from this closely related cell population.

Interestingly, inspection of serial brainstem sections from these same mice after Introvert PRV was injected into the lumbar spinal cord indicated that 4.2 ± 2.1 (SD) viral infected cells could be found in the ventral aspect of the reticular nucleus gigantocellularis (*n* = 3 mice, Figure 1C), a brainstem region housing glutamatergic neurons involved in generating high speed locomotor activity (Capelli et al., 2017), as well as glycinergic neurons that result in the cessation of stepping (Bouvier et al., 2015). Although we were not able to determine whether excitatory or inhibitory neurons within this region contact the WT1-expressing population, our data does raise the possibility that the reticular nucleus may provide ongoing modulation of the activity of interneuronal components of this neural network, circuitry that would be functionally relevant when quadrupedal animals switch between gaits from walking to trotting to galloping.

## Interneuronal Populations Potentially Involved In Locomotor Rhythm Generation

The transcription factor Shox2 is expressed in a subset of V2a neurons as well as other, Chx10 negative interneurons. These Shox2+/Chx10− neurons have recently been shown to be excitatory, and possess many properties of locomotor rhythm generating neurons (Dougherty et al., 2013). As a whole (i.e., no distinction could be made between Chx10+ and Chx10- subsets), Shox2-expressing neurons project toward ipsilateral targets including other Shox2+ interneurons, motoneurons, as well as unidentified commissural interneurons (Dougherty et al., 2013). Interestingly in depth analysis of the projection pattern of Shox2 neurons that were Chx10+ and Chx10- indicates that each subset only projects to other members belonging to the same subtype and there is no connectivity between Chx10+ and Chx10- Shox2+ neurons (Ha and Dougherty, 2018).

Another population of interneurons located in close proximity to the Shox2+ population is marked by postnatal expression of Hb9 (Hinckley et al., 2005). Members of this small population of excitatory interneurons, project to one another via chemical and electrical synapses as well as onto ipsilateral motoneurons (Hinckley and Zisnind-Conhaim, 2006; Wilson et al., 2007), and they have also been implicated in locomotor rhythm generation (Brownstone and Wilson, 2008; Hinckley et al., 2010; Caldeira et al., 2017), although their involvement has been debated (see Kwan et al., 2009).

## Discussion

We are now approaching 20 years since the first published work incorporating a molecular genetic approach to identify, and functionally characterize, interneuronal components of the locomotor CPG. While this approach has resulted in a tremendous amount of new data, we still know little regarding the manner in which these populations interact with one another. This information is required if we hope to understand how this neural circuit operates, and devise therapies targeted at restoring function after spinal cord injury.

Thus far a number of studies have used approaches such as the retrograde transynaptic transport of PRV-152 to identify interneuronal populations that contact hindlimb motoneurons, however relatively little connectivity between the genetically-defined interneuronal populations has been revealed. While it is certainly valuable to identify last order interneurons that project to hindlimb motoneurons, it is essential to keep in mind that these experiments do not indicate exclusivity, and that these last order interneurons are also likely to project to other interneuronal populations. In fact, each of the studies that have incorporated an anterograde approach to investigate the distribution of axon terminals of an interneuronal population has led to the identification of input to multiple regions, or onto multiple cell types, within the spinal cord (see Table 1). Given the vast diversity within each parent population these findings are not surprising. While this makes studies into the connectivity of the locomotor CPG more technically demanding, recent progress in the development of viral approaches (reviewed in Luo et al., 2018) is beginning to provide the tools required to reveal the manner in which these genetically-defined interneuronal populations are activated and interconnected. As more discrete subsets of interneurons are identified within each parent population, these techniques are likely to become increasingly valuable for deciphering connectivity amongst them.

**TABLE 1:** Axonal projection pattern and identified synaptic targets for each of the genetically-defined interneuronal populations located in the ventral spinal cord postnatally that have been identified to participate in locomotor activity.

**Population**	**Subset**	**Transmitter**	**Axonal projection**	**Synaptic targets**
V0	V0D	Inhibitory	Contralateral	Contralateral motoneurons
	V0V	Excitatory	Contralateral	
	V0C	Excitatory	Ipsi/contralateral	ipsi/contralateral motoneurons, unidentified lamina VIII and dorsal horn interneurons
V1	RC	Inhibitory	Ipsilateral	Motoneurons, IaINs
	IaIN	Inhibitory	Ipsilateral	Motoneurons
	V1	Inhibitory	Ipsilateral	Flexor motoneurons, unidentified ventral interneurons
V2	V2a	Excitatory	Ipsilateral	V0_V_ and unidentified commissural interneurons
	V2b	Inhibitory	Ipsilateral	Extensor motoneurons, V0c INs, unidentified neurons in lamina VII and VIII
V3		Excitatory	Ipsi/contralateral	Ipsi/contralateral motoneurons, Renshaw cells, IaINs, ipsilateral V3 interneurons as well as unidentified ipsilateral and contralateral targets.
dI6	DMRT3	Inhibitory	Ipsi/contralateral	Ipsilateral and contralateral motoneurons
	WT1	Inhibitory	Ipsi/contralateral	DMRT3 + dI6 cells and V0V interneurons

## Data Availability Statement

The datasets generated for this study are available on request to the corresponding author.

## Ethics Statement

The animal study was reviewed and approved by IACUC- University of Alberta.

## Author Contributions

Both authors listed have made a substantial, direct and intellectual contribution to the work, and approved it for publication.

## Conflict of Interest

The authors declare that the research was conducted in the absence of any commercial or financial relationships that could be construed as a potential conflict of interest.
